# Supplementary material for: Sleep Characteristics in Individuals with Ehlers-Danlos Syndrome
Source: Med Sci (Basel). 2025 Jun 27;13(3):85. doi: 10.3390/medsci13030085 (PMC12286137; doi:10.3390/medsci13030085)
Supplement: Supplementary file 1 [file medsci-13-00085-s001.zip › medsci-3673970-supplementary.pdf]

Q

Characteristics of Sleep and Sleep Disorders in Individuals with Ehlers-Danlos Syndrome (EDS)

This study is being conducted to examine the sleep patterns, sleep disturbances, and interventions used to improve sleep for individuals with EDS. We appreciate your time in taking this survey and our goals are to utilize this information in the development of new research to address sleep disorders. Thank you!

Block 1

Add Question

INFORMED CONSENT

Ehlers-Danlos Syndrome and Sleep
You are being invited to participate in a research study about sleep and Ehlers-Danlos Syndrome. This study is being conducted at the University of Tennessee at Chattanooga (UTC) by David Levine (david-levine@utc.edu). The questionnaire(s) will take about 10 minutes to complete.
There are no foreseeable risks or direct benefits to you if you choose to participate in this study. The information gained from this research may benefit others in the future.
This survey is anonymous. Do not include your name or any of your contact information in your responses to the survey. Your responses to the survey will not be linked to your computer, email address or other electronic identifiers. No one will be able to identify you or your answers.
Your participation in this study is voluntary. You are free to stop answering questions at any time or to decline to answer any question you do not wish to answer for any reason. If you stop the survey before the end, your previous answers will be automatically discarded. After you submit the survey, we cannot remove your responses because we will not know which answers came from you.
Research at UTC involving human participants is carried out under the oversight of the Institutional Review Board. Address questions or problems regarding these activities to Dr. Susan Davidson, UTC IRB Chair, email: susan-davidson@utc.edu; phone: (423) 425-1387.
Please indicate your decision regarding participation in this research by selecting a response below: *

- ◯ I am 18 years of age or older, have a diagnosis of Ehlers-Danlos Syndrome, and consent to voluntarily participate in this survey.
- ◯ I do not consent or do not have Ehlers-Danlos Syndrome Terminate

Add Question Automatic Logic Break Separator Split Block

What is your gender?

- ◯ Female
- ◯ Male
- ◯ Prefer not to answer

Add Question Page Break Separator Split Block

Age:

- ◯ 18-24
- ◯ 25-34
- ◯ 35-44
- ◯ 45-54
- ◯ 55-64
- ◯ 65-74
- ◯ 75+

Add Question Page Break Separator Split Block

What is your race?

- American Indian or Alaska Native
- Asian
- Black or African American
- Caucasian or White
- Native Hawaiian or Other Pacific Islander
- Other
- Prefer not to answer

Add Question Page Break Separator Split Block

What is your ethnic background? (e.g. Ashkenazi Jew, Hispanic or Latino, African American, Norwegian)

Answer text

Add Question Page Break Separator Split Block

What is your primary country of residence?

Answer text

Add Question Page Break Separator Split Block

What type of EDS do you have?

- Classical EDS (cEDS)
- Hypermobile EDS (hEDS)
- Classical-like EDS (clEDS)
- Cardiac-valvular EDS (cvEDS)
- Vascular EDS (vEDS)
- Arthrochalasia EDS (aEDS)
- Dermatosparaxis EDS (dEDS)
- Kyphoscoliotic EDS (kEDS)
- Brittle Cornea Syndrome (BCS)
- Spondylodysplastic EDS (spEDS)
- Musculocontractural EDS (mcEDS)
- Myopathic EDS (mEDS)
- Periodontal EDS (pEDS)
- Unsure

Add Question

Page Break Separator

Block 1

Add Block Merge Block

Block 2

Add Question

On average, how many hours of sleep do you get in a typical **24 hour period** (including naps)?

- 0-5.99 hours
- 6-7.99 hours
- 8-9.99 hours
- 10-11.99 hours
- 12+

Add Question

Page Break Separator

Block 2

Add Block Merge Block

Block 3 - PROMIS Sleep

Add Question

In the past 7 days... (PROMIS Sleep Disturbance, 2021)

	Left Anchor				Right Anchor
	Not at all	A little bit	Somewhat	Quite a bit	Very much
my sleep was restful	◯	◯	◯	◯	◯
my sleep was light	◯	◯	◯	◯	◯
my sleep was deep	◯	◯	◯	◯	◯
my sleep was restless	◯	◯	◯	◯	◯
I was satisfied with my sleep	◯	◯	◯	◯	◯
	Left Anchor				Right Anchor
my sleep was refreshing	◯	◯	◯	◯	◯
	Not at all	A little bit	Somewhat	Quite a bit	Very much
I felt lousy when I woke up	◯	◯	◯	◯	◯

I had a problem with my sleep	○	○	○	○	○
I had difficulty falling asleep	○	○	○	○	○
I felt physically tense at bedtime	○	○	○	○	○
I worried about not being able to fall asleep	○	○	○	○	○
I felt worried at bedtime	○	○	○	○	○
I had trouble stopping my thoughts at bedtime	○	○	○	○	○
I felt sad at bedtime	○	○	○	○	○
I had trouble getting into a comfortable position to sleep	○	○	○	○	○
I tried hard to get to sleep	○	○	○	○	○
stress disturbed my sleep	○	○	○	○	○
I tossed and turned at night	○	○	○	○	○
I was afraid I would not get back to sleep after waking up	○	○	○	○	○

Add Question

Page Break Separator Split Block

In the past 7 days...

	Left Anchor				Right Anchor
	Never	Rarely	Sometimes	Often	Always
I got enough sleep	○	○	○	○	○
It was easy for me to fall asleep	○	○	○	○	○

Left Anchor Right Anchor
Never Rarely Sometimes Often Always

I laid in bed for hours waiting to fall asleep	○	○	○	○	○
I woke up too early and could not fall back asleep	○	○	○	○	○
I had trouble staying asleep	○	○	○	○	○
I had trouble sleeping	○	○	○	○	○
I woke up and had trouble falling back to sleep	○	○	○	○	○

Add Question

Page Break ☐ Separator Split Block

In the past 7 days...

	Left Anchor				Right Anchor
	Very poor	Poor	Fair	Good	Very good
my sleep quality was	○	○	○	○	○

Add Question

Page Break ☐ Separator

Block 3 - PROMIS Sleep

Add Block Merge Block

Block 4

10 Questions

Add Question

How long does it take you to fall asleep at night?

- 0-15 minutes
- 16-30 minutes
- 31-45 minutes
- 46-60 minutes
- 61-90 minutes
- 91-120 minutes
- More than 120 minutes

Add Question Page Break Separator Split Block

In the past 30 days, how often have you had trouble sleeping because you had to get up to use the restroom:

- Never
- Less than once a week
- Once or twice a week
- Three or four times a week
- Five or six times a week
- Every day

Add Question Page Break Separator Split Block

In the past 30 days, how often have you had trouble sleeping because you could not breathe comfortably?

- Never

- Less than once a week
- Once or twice a week
- Three or more times a week
- Five or six times a week
- Every day

Add Question

Page Break   Separator   Split Block

In the past 30 days, how often have you had trouble sleeping because you were too cold?

- Never
- Less than once a week
- Once or twice a week
- Three or four times a week
- Five or six times a week
- Every day

Add Question

Page Break   Separator   Split Block

In the past 30 days, how often have you had trouble sleeping because you were too hot?

- Never
- Less than once a week
- Once or twice a week
- Three or four times a week

- Five or six times a week
- Every day

Add Question Page Break Separator Split Block

In the past 30 days, how often have you had trouble sleeping because you were in pain?

- Never
- Less than once a week
- Once or twice a week
- Three or four times a week
- Five or six times a week
- Every day

Add Question Page Break Separator Split Block

During the past 30 days, how much has your fatigue interfered with your everyday activities?

- Never
- Less than once a week
- Once or twice a week
- Three or four times a week
- Five or six times a week
- Every day

Add Question

Page Break Separator Split Block

In the past 30 days, how much did pain interfere with your everyday activities?

- Never
- Less than once a week
- Once or twice a week
- Three or four times a week
- Five or more times a week
- Every day

Add Question

Page Break Separator Split Block

Now thinking about your physical health, which includes physical illness and injury, for how many days **during the past 30 days** was your physical health **not good?** Please respond with a number from 0 to 30.

Answer text

Add Question

Page Break Separator Split Block

Now thinking about your mental health, which includes stress, depression, and problems with emotions, for how many days **during the past 30 days** was your mental health **not good?** Please respond with a number from 0 to 30.

Answer text

Add Question

Page Break Separator

Block 4

10 Questions

Add Block Merge Block

Block 4

Add Question

What helps you fall asleep? (Please list anything that helps you fall asleep).

Answer text

Add Text Box

Add Question

Page Break Separator Split Block

What assistive devices/equipment do you use to fall asleep?

- ☐ weighted blanket
- ☐ eye mask
- ☐ ear plugs
- ☐ white noise machine
- ☐ positioning/pillows or wedges
- ☐ lighting (i.e. blackout , use a night light, etc.)
- ☐ room temperature
- ☐ calming/mindfulness phone applications
- ☐ clothing

- ☐ cognitive behavioral therpay
- ☐ other, please specify.
- ☐ none

Add Question Page Break Separator Split Block

Do you take any over the counter medication to help you sleep?

- ○ Yes
- ○ No

Add Question Page Break Separator Split Block

Do you take any prescription medications to help you fall asleep?

- ○ Yes
- ○ No

Add Question Page Break Separator Split Block

Do you have a sleep routine/schedule (i.e. go to bed and wake up at the same time everyday)?

- ○ Yes
- ○ No

Add Question — Page Break — Separator — Split Block

Have you ever been diagnosed with a sleep disorder?

- Yes
- No

Add Question — Page Break — Separator — Split Block

Have you ever been diagnosed with sleep apnea?

- Yes
- No

Add Question — Page Break — Separator — Split Block

Do you use a CPAP machine?

- Yes
- No

Add Question — Page Break — Separator

Block 4

Add Block

Edit Footer Thank You Page

University License - Research Edition
©2022 QuestionPro
